# Characteristics and significance of fever during 4 weeks after primary total knee arthroplasty

**DOI:** 10.1007/s00402-014-1949-0

**Published:** 2014-02-13

**Authors:** Yoshinori Ishii, Hideo Noguchi, Mitsuhiro Takeda, Junko Sato, Satoshi Takayama, Shin-ichi Toyabe

**Affiliations:** 1 Ishii Orthopaedic and Rehabilitation Clinic, 1089 Shimo-Oshi, Gyoda, Saitama 361-0037 Japan; 2 Division of Information Science and Biostatistics, Niigata University Graduate School of Medical and Dental Sciences, 1 Asahimachi Dori Niigata, Niigata, 951-8520 Japan

**Keywords:** Total knee arthroplasty, Postoperative fever, Maximum temperature, Periprosthetic infection

## Abstract

**Purpose:**

Most previous studies on postoperative fever (POF; ≥38 °C) after total knee arthroplasty (TKA) have reported findings from only the immediate postoperative days (PODs). The hypothesis of the current study is that 4 weeks of follow-up may reveal differences in the characteristics of POF and fever-related factors between a normal inflammatory response and an early acute infection-related response.

**Methods:**

A total of 400 consecutive TKAs (314 patients) were retrospectively investigated. Patients were stratified into those who developed an early acute periprosthetic infection that required subsequent surgical treatment (STG; *n* = 5 TKAs) and those who did not (non-STG; *n* = 395 TKAs).

**Results:**

Among the 400 knees, 149 (37 %) developed POF, with most reaching a maximum temperature (MT) on POD 0. In 13 TKA patients who had POF with a peak daily temperature ≥38 °C during postoperative weeks 2–4, the causes of POF were respiratory and urinary tract infections (*n* = 5 for each), superficial infection (*n* = 2), and periprosthetic infection (*n* = 1). The STG and non-STG differed significantly with regard to the rate of POF (*p* = 0.0205) and MT (*p* = 0.0003), including MTs less than 38 °C, during postoperative weeks 2–4. All five STG patients had elevated C-reactive protein levels and local symptomatic findings before the additional surgery.

**Conclusions:**

The occurrence of POF and MT along with elevated C-reactive protein and local symptomatic findings at 2–4 weeks postoperatively may indicate the need for a positive fever workup to recognize early acute periprosthetic infection.

## Introduction

Although fever is commonly associated with infection, postoperative fever (POF), defined as a body temperature ≥38 °C (100.4 °F) in the postoperative period, may be a normal physiological response after total knee arthroplasty (TKA). POF is experienced in 40–50 % of patients who undergo total joint arthroplasty (TJA) [1]. An increased temperature may occur within the first 5 days following hip or knee replacement [1–6]. Most authors have concluded that a sepsis workup is unnecessary if the temperature progressively decreases, and the decision should be based on physical examination and symptoms. Conversely, infection after TJA imposes clinical and financial burdens in terms of both diagnosis and treatment of periprosthetic sepsis [6–8]. Prompt diagnosis of periprosthetic infection in TJA patients permits early treatment, which is particularly important for acute infections occurring within the first 4 [9] or 6 weeks [8]. In addition, considering the 30-day mortality period, reports from the Swedish Knee Arthroplasty Register [10] suggest that it is safer to operate on one knee at a time, rather than on two knees simultaneously. To address complications in the early period after TKA (e.g., the first 4–6 weeks), surgeons and primary care physicians must properly assess the requirement for further workup.

Most previous studies on POF after TKA have reported findings from only the immediate postoperative days (PODs) of a patient’s hospital stay [1–4, 11]. The Japanese National Medical Insurance system allows patients to determine, to some degree, the length of their hospital stay given reasonable justification such as uncontrolled pain, unstable transfer, or other perioperative complications. For 345 TKA patients in Japan, Yasunaga et al. [12] reported an average hospital stay of 35.1 ± 15.9 days. Mitsuyasu et al. [13] reported mean stays of 39.24 ± 18.09 and 54.44 ± 26.60 days after TKA in high- and low-volume hospitals, respectively. Because the average hospital stay after TKA in our institute is 41 days [14], it was possible for us to investigate the characteristics of POF for a longer time period than in previous reports. We were also able to compare various factors affecting POF and indicators of infection such as decreased hematocrit (Ht) [15], transfusion [15, 16], leukocytosis [17], and an elevated serum C-reactive protein (CRP) level [17, 18] over a longer time period than in previous studies.

The purpose of the present study was to describe the incidence and characteristics of POF after TKA and to clarify the significance of the febrile response in terms of periprosthetic infection and febrile-related factors for a longer period (4 weeks) than in previous studies. Our hypothesis was that a 4-week follow-up period may reveal differences in the characteristics of POF and fever-related factors between a normal inflammatory response and an early acute infection-related response, defined as infection occurring in the first 4 weeks postoperatively [9].

## Materials and methods

Our institutional review board approved this retrospective review of medical records and the analysis of pertinent data from patients treated with primary TKA from 1998 to 2012. A total of 314 patients (400 TKAs) were included in this study. The preoperative diagnosis for TKA was osteoarthritis in 388 knees and rheumatoid arthritis in 12 knees. All patients with bilateral disease were scheduled to undergo staged bilateral TKA. The average follow-up period was 91 months (range, 6–184 months). The clinical characteristics of the patients are summarized in Table 1. These patients were stratified into two groups: those who developed an early acute periprosthetic infection that required surgical treatment within 4 weeks after TKA (STG; *n* = 5 TKAs), and those who did not (non-STG; *n* = 395 TKAs). No patients except these five patients developed deep infection during the follow-up period.

**Table 1:** Patient characteristics

Characteristic	Value
Number of patients	314 patients (400 knees)
Mean age	72 ± 8 years
Gender	Male, 53 TKAs; female, 347 TKAs
Preoperative diagnosis	OA, 388 TKAs; RA, 12 TKAs
Body height	151 ± 7 cm
Body weight	59 ± 11 kg
BMI	26 ± 4
Follow-up period	91 ± 50 months

*TKA* total knee arthroplasty, *OA* osteoarthritis, *RA* rheumatoid arthritis

### Perioperative treatment protocol

During surgery, 400 ml of autogenous blood that had been preserved for 1 week preoperatively was infused into each patient. A CBCII ConstaVac Blood Conservation System (Stryker, Kalamazoo, MI) was used in all patients after surgery. The volume of blood collected was returned to the patients within 6 h of surgery. A foot pump (A-V Impulse System; Novamedix, Andover, UK) was used to prevent deep venous thrombosis. The drains were removed routinely during the first dressing change on POD 1, after which the patients were allowed full weight-bearing ambulation. All patients received perioperative antibiotics for prophylaxis and analgesics for pain. No patients received medication for thromboembolism.

### Definitions

All patients’ charts were evaluated for POF for 4 weeks of their hospital stay. Axillary temperatures were recorded using standard digital thermometers every hour on the day of surgery and then every 8 h according to a specific nursing care map until 6:00 a.m. the following day. POD 1 began at 6:00 a.m. the day after surgery and continued for 24 h. Subsequent PODs began and ended at 6:00 a.m. in a repeating sequence. For patients who underwent bilateral procedures during different hospitalization periods, each surgical procedure and postoperative course was analyzed separately (no simultaneous bilateral procedures were performed). Perioperative days were defined as reported by Summersell et al. [5] and modified as follows: the day before the operation was day −1 (POD − 1), which was also called the preoperative baseline; the day of surgery was day 0 (POD 0); the first day after surgery was day 1 (POD 1); and so on. The peak daily temperature (PDT) was defined as the highest temperature recorded on a given day for each patient. The maximum temperature (MT) was the highest PDT recorded for each patient during the study period.

In patients who developed fever, the MT and PDT were appropriately recorded. Serological factors that reportedly affect the febrile response, including WBC count [2, 9], Ht [15], and CRP level [8, 18], were routinely determined to monitor for bacterial infection [18] and to assess the degree of intraoperative tissue damage [20] at postoperative weeks 1 and 4. Leukocytosis was defined as a WBC count ≥9,000/ml. Estimated blood loss was calculated using the maximum decrease in the Ht measured 1 week after surgery and normalized to the patient’s weight and height according to Gross [21].

### Statistical analysis

Differences between the STG and non-STG were analyzed using Wilcoxon’s rank sum test and Fisher’s exact test. Statistical analyses were performed using IBM SPSS Statistics ver. 20 (IBM Japan, Tokyo, Japan). In all tests, a *p* value <0.05 was considered to indicate statistical significance.

## Results

The maximum mean PDT was 37.5 °C on the day of TKA surgery (Table 2). A total of 149 knees (37 %) developed POF, most with a MT on POD 0 (Table 3). Of these 149 knees, 142 experienced a MT within 1 week postoperatively, and seven did so in the subsequent 3 weeks (Table 3). In 13 TKA patients who had POF with a peak daily temperature ≥38 °C during postoperative weeks 2–4, the causes of POF were respiratory and urinary tract infections (*n* = 5 for each), superficial infection (*n* = 2), and periprosthetic infection (*n* = 1). (Table 2). The respiratory and urinary cases had no local symptoms around the knee, including no pain, swelling, warmth, or inflammation.

**Table 2:** Mean peak daily temperature during follow-up period

	Postoperative day											
Non-STG group	−1	0	1	2	3	4	5	6	7	8–14^a^	15–21^a^	22–28^a^
Mean PDT (SD)	36.2 (0.5)	37.5 (0.5)	37.4 (0.6)	37.2 (0.6)	36.9 (0.6)	36.7 (0.6)	36.6 (0.6)	36.5 (0.6)	36.4 (0.6)	36.8 (0.4)	36.7* (0.4)	36.7** (0.4)
No. with POF (PDT ≥38 °C) 219 knees, 146 patients	0	79	65	34	13	1	7	4	4	6	4	2
STG group												
Mean PDT (SD)	36.0 (0.4)	37.5 (0.7)	37.4 (0.6)	36.8 (0.4)	36.9 (0.5)	36.8 (0.3)	36.6 (0.2)	36.5 (0.5)	36.5 (0.2)	37.0 (0.5)	37.4* (0.6)	37.1** (0.6)
No. with POF (PDT ≥38 °C) 4 knees, 3 patients	0	1	1	0	0	0	0	0	0	0	1	1

*STG* surgical treatment, *PDT* peak daily temperature, *POF* postoperative fever

^a ^Mean PDT during each week

* *p* = 0.001

** *p* = 0.008

**Table 3:** Postoperative day of maximum temperature

	Postoperative Day											
Non-STG group	−1	0	1	2	3	4	5	6	7	8–14	15–21	22–28
Days of MT (*N* = 395)	0	195	111	55	14	3	4	1	1	5	3	3
No. with MT ≥38 °C (*N* = 146)	0	66	43	22	5	0	2	1	1	1	3	2
STG group												
Days of MT (*N* = 5)	0	1	1	0	0	0	0	0	0	0	3	0
No. with MT ≥38 °C (*N* = 3)	0	1	1	0	0	0	0	0	0	0	1	0

*STG* surgical treatment, *MT* maximum temperature

During post-TKA weeks 2–4, five knees in five patients developed early acute infection as indicated by massive discharge or joint swelling with elevated CRP (median, 10.5 mg/l; range, 12.7–23.1). These cases required additional surgical procedures on median POD 21 (range, 14–74). Four knees (primary causative organisms: *Staphylococcus aureus*, 2 cases; *Enterococcus faecalis*, 1 case; and *Candida* and *Enterococcus* spp., 1 case) required massive irrigation, meticulous joint debridement or synovectomy, and one knee infected with methicillin-resistant *S. aureus* (MRSA) required the removal of components.

There were significant differences in the PDT between the STG and non-STG during post-TKA weeks 3 and 4 (POD 15–21, *p* = 0.001; POD 22–28, *p* = 0.008), but there were no obvious differences during week 1 (Table 2). In two of the five knees in the STG, the MT occurred on POD 0 and POD 1, respectively (Tables 2, 3), and in three of the five STG knees, the MT occurred during postoperative weeks 2–4. However, during this period, only the MRSA-infected case among these three knees had a MT ≥38 °C; the other two infected cases had a MT <38 °C (Table 3). Thus, only the MRSA-infected case among the five STG patients had POF (MT ≥38 °C) during postoperative weeks 2–4.

The rates of occurrence of POF (STG, 2/4; non-STG, 12/219, Table 2) and of MT (STG, 3/5; non-STG, 11/395, Table 3) during postoperative weeks 2–4 were significantly different between the two groups (*p* = 0.0205 and *p* = 0.0003, respectively). There were no differences in perioperative fever-related factors between the STG and non-STG (Table 4), although a statistical type II error should be taken into account because of the small number of non-STG cases. In addition, the RBC count, Ht, and CRP level at 4 weeks post-TKA differed significantly between the two groups, but no differences were observed at 1 week (Table 5). No cases in this series experienced postoperative leukocytosis.

**Table 4:** Comparison of perioperative fever-related factors between the surgical (STG) and non-surgical (non-STG) treatment groups

Factor	Non-STG (*N* = 395)	STG (*N* = 5)	*p* value
Patients age^a^	72 (8)	77 (4)	0.082
Gender (male/female)^b^	52/343	1/4	0.511
BMI^a^	26 (4)	28 (5)	0.438
Operation time^a^	60 (14)	58 (10)	0.972
Tourniquet time^a^	59 (13)	56 (11)	0.480
Estimate blood loss^a^	956 (441)	1,071 (457)	0.660
Auto-transfusion volume^a^	338 (209)	400 (76)	0.410
Anesthesia (General/Spinal)^b^	236/159	4/1	0.652

Values are presented as average (SD) or number

*BMI* Body mass index

^a ^Wilcoxon rank sum test

^b ^Fisher exact test

**Table 5:** Comparison of serological data between the surgical (STG; *N* = 5) and non-STG (*N* = 395) treatment groups during 4 weeks after surgery

Parameter	Preoperative		1 week postoperatively		4 weeks postoperatively	
Average (SD)	Non-STG	STG	Non-STG	STG	Non-STG	STG
WBC	6,003 (1,766)	6,600 (2,666)	5,824 (1,628)	5,340 (1,071)	5,287 (1,507)	6,060 (918)
RBC	426 (40)	414 (40)	323 (43)	302 (31)	379 (38)*	326 (43)*
Ht (%)	39.9 (3.8)	40.5 (2.6)	30.7 (4.3)	30.3 (2.8)	36.1 (3.9)**	31.9 (4.1)**
CRP (mg/l)	0.43 (1.03)	0.15 (0.10)	2.39 (1.87)	2.06 (0.59)	0.53 (1.36)***	2.27 (2.08)***

*WBC* white blood cell count, *RBC* red blood cell count, *Ht* hematocrit, *CRP* C-reactive protein

* *p* = 0.011, ** *p* = 0.027, *** *p* = 0.010

## Discussion

The most important finding of this study was that in 37 % of TKAs, the patients experienced POF, with most developing a MT on POD 0, followed by POD 1. The temperature gradually returned to normal by POD 7 in STG and non-STG patients, which is in agreement with previous studies reporting a return by POD 6 [1, 3, 6]. The rate of occurrence of POF in the present study was within the previously reported range of values, while the POD of the MT was earlier than those in previous reports [1–3, 6, 11, 15, 19]. In earlier studies, approximately 8.4–68 % of patients who underwent TKA developed a fever, with most developing a MT on POD 2 [1–3, 6, 11, 15] or POD 1 [4, 19]. In addition, the occurrences of POF and MT (including MTs <38 °C) in weeks 2 through 4 after TKA in the STG were higher than those in the non-STG, suggesting that this may be a predictor of additional surgery because of early acute periprosthetic infection (defined as infection occurring within the first 4 weeks after surgery).

Regarding the correlation between the febrile pattern and infection within the first week after TKA, Ward et al. [6] reported that fever occurring after POD 3 and multiple febrile days were independent predictors of a positive workup and that patients with a MT ≥39.0 °C had a significantly higher rate of fever. The present results indicate that the appearance of POF and MT during weeks 2–4 postoperatively may necessitate a fever workup to detect potential early acute periprosthetic infection. However, this decision should be based on physical examination and local symptoms. In fact, the current study revealed that no patients without local symptoms who had a POF (≥38 °C) during the 4 weeks after TKA suffered from acute periprosthetic infection. In addition, although none of the four infected cases (excluding the MRSA-infected case) had POF in postoperative weeks 2–4, they did exhibit local symptoms such as pain, swelling, warmth, inflammation, and discharge.

With regard to the perioperative fever-related factors and postoperative serological investigations at week 1, we were unable to distinguish a difference between the STG and non-STG cases. The five STG patients had an elevated CRP level before additional surgery was performed on average POD 18. The RBC count, Ht, and CRP levels at 4 weeks after TKA were significantly different between the two groups. The significantly lower RBC and Ht and higher CRP levels in the STG may represent secondary reactions to infection and different degrees of inflammation compared with the non-STG. Because the WBC count did not differ between 1 and 4 weeks postoperatively, it is unlikely to be an important indicator of early acute periprosthetic infection. Toossi et al. [22] also recently concluded that the serum WBC count and differential counts do not have a role in the diagnosis of periprosthetic joint infection. Nevertheless, we recognize that this result is not conclusive because the sample size of the non-STG was relatively small.

Finally, it is critical for surgeons and primary care physicians who are experienced in the management of medical problems in hyperthermic patients to properly judge the necessity of a further workup for identifying infection. Fevers may develop due to septic or aseptic causes such as pulmonary embolism, pneumonia, urinary tract infection, deep venous thrombosis, surgical wound infection, or intravenous phlebitis. POF has also been linked to a transient disturbance of temperature regulation after general anesthesia, surgery, and pyrogens (both endogenous and exogenous) acting on the nervous system [1–4, 6, 11, 15, 19, 23, 24]. Considering that the appearance of POF and MT during postoperative weeks 2–4 may indicate the need for additional surgical treatment after TKA, this may help surgeons and primary care physicians to distinguish a normal postoperative physiological febrile response from a periprosthetic infection-related response when physical examination or symptoms coincide with infection. In addition, the current study can help to prevent unnecessary sepsis workups and possible delays in discharge, and patients may thus confine their visits to primary care physicians during the first 4 weeks after surgery when they are determined not to have an early acute infection. As a result, the overall cost of TKA could be decreased.

Our study had some limitations. First, it was a retrospective analysis. Second, the patients in this study were treated with non-steroidal anti-inflammatory drugs for pain control, which might have attenuated the febrile response in these patients. Third, all patients received antibiotics from POD 0 to 3, and this might also have decreased the febrile response. Fourth, although we acknowledge the release or increase of pyogenic mediators such as interleukin (IL)-6, IL-1β, and tumor necrosis factor-α in both the serum and discharge of patients who underwent TJA [19, 23], the autogenous blood collected with the blood conservation system was returned to the patients within 6 h of surgery. We did not evaluate pyogenic mediators because of the cost and difficulty of doing so in a busy clinical atmosphere. An advantage of this study is that the fever pattern was analyzed during a longer period after TKA (4 weeks) than in previous studies [1, 2, 4, 6], which were undertaken by one experienced surgeon using the same protocol.

In conclusion, POF is common and may be part of a normal inflammatory response to tissue injury after TKA. More than one-third of patients often experience POF within 4 weeks after TKA, with most developing a MT on POD 0. The appearance of POF and MT (including <38 °C) during post-TKA weeks 2–4 may indicate the necessity of a fever workup to rule out periprosthetic infection. In addition to the CRP level, physical examination, and monitoring for local symptoms, 4 weeks of follow-up due to a febrile response after TKA may be cost-effective (patients can also check their temperature at home) and necessary to detect early acute periprosthetic infection.
